# Nano-Medicine in the Cardiovascular System

**DOI:** 10.3389/fphar.2021.640182

**Published:** 2021-03-04

**Authors:** Danielle Pretorius, Vahid Serpooshan, Jianyi Zhang

**Affiliations:** ^1^ Department of Biomedical Engineering, School of Medicine, University of Alabama at Birmingham, Birmingham, AL, United States; ^2^ Department of Biomedical Engineering, School of Engineering, University of Alabama at Birmingham, Birmingham, AL, United States; ^3^ Emory Children’s Center, Emory University School of Medicine, Atlanta, GA, United States

**Keywords:** cardiac nano-medicine, therapeutic nanoparticles, diagnostic nanoparticles, regenerative exosomes, cardio-protective nanoparticles

## Abstract

Nano-medicines that include nanoparticles, nanocomposites, small molecules, and exosomes represent new viable sources for future therapies for the dysfunction of cardiovascular system, as well as the other important organ systems. Nanomaterials possess special properties ranging from their intrinsic physicochemical properties, surface energy and surface topographies which can illicit advantageous cellular responses within the cardiovascular system, making them exceptionally valuable in future clinical translation applications. The success of nano-medicines as future cardiovascular theranostic agents requires a comprehensive understanding of the intersection between nanomaterial and the biomedical fields. In this review, we highlight some of the major types of nano-medicine systems that are currently being explored in the cardiac field. This review focusses on the major differences between the systems, and how these differences affect the specific therapeutic or diagnostic applications. The important concerns relevant to cardiac nano-medicines, including cellular responses, toxicity of the different nanomaterials, as well as cardio-protective and regenerative capabilities are discussed. In this review an overview of the current development of nano-medicines specific to the cardiac field is provided, discussing the diverse nature and applications of nanomaterials as therapeutic and diagnostic agents.

## Introduction

Nano-medicines have shown great promise various cardiac applications due to their unique and characteristic properties. In stark contrast to bulk implants, nanomaterials present the capacity to be mobile in both intra- and extra-vascular systems, making them ideal cargo delivery systems and/or potential imaging agents. When designing a delivery system, it is not only important to consider what the intended load will be, but also what type of material will be utilized. Material-chemistry affects the physical properties of the system, and subsequently the system’s performance ability.

Nanomaterials have demonstrated great potential for cardiovascular medicine applications due to their ability to be utilized for multiple purposes. Nanostructured surfaces have the ability to, via topographical cues, control and selectively direct cell activities (Park et al., 2007; Brammer et al., 2008; Oh et al., 2009; Pan et al., 2012). This ability to selectively guide cellular activity is one that can be very useful in engineered approaches to where the activity of one cell type needs to be suppressed, while the activity of another cell type needs to be promoted. By coating a coronary stent with the proper nanostructured surface could potentially suppress the growth of smooth muscle cells (SMCs), while encouraging the attachment and proliferation of endothelial cells (ECs) (Serruys et al., 2006).

In this review, we highlight some of the major types of nano-medicine systems that are currently being explored in the cardiac field. With special attention given to the major differences between the systems, and how these differences affect the specific therapeutic or diagnostic applications of the systems. The important concerns relevant to cardiovascular nano-medicines, such as cellular responses, toxicity of nanomaterials, as well as cardio-protective and regenerative capabilities are discussed. This review provides an overview of the current development of nano-medicines being developed for use in the cardiac field, while displaying the diverse nature and applications of nanomaterials as therapeutic and diagnostic agents.

## Types of Scaffold in Nano-Medicine

Nano-medicines, whether fabricated for therapeutic or diagnostic, or both [theranostic (Kelkar and Reineke, 2011)] purposes can consist of organic or inorganic substrates. Organic substrates, for the purposes of this review are defined as those consisting of mostly a carbon backbone, with additional hydrogen, oxygen or nitrogen covalently bound to it. Inorganic substrates on the other hand, include salts, metal oxides and metal frameworks for example, but more specifically, they are compounds that do not contain the (-CH-) bonds associated with organic compounds (see Table 1 for a brief summary). The type of material utilized in the fabrication of nano-medicines is greatly affected by the application of the system. The following section will delve into some of the prevalent materials that have been explored and utilized in cardiac nano-medicines, specifically considering the benefits and potential drawbacks of each.

**TABLE 1:** Short summary showing the differences between organic and inorganic compounds.

Organic compounds	Inorganic compounds
Contains carbons and hydrogens (-CH-) groups	No (-CH-) groups
Covalent bonds	Ionic and covalent bonds
Examples include: 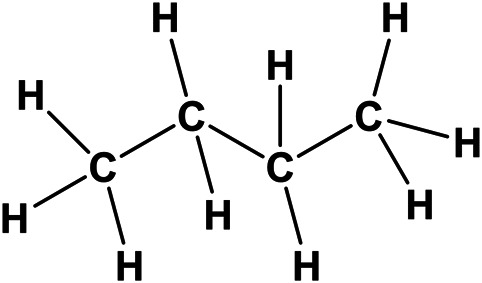	Examples include: 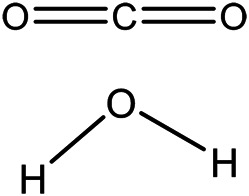

### Organic Scaffolds

**FIGURE 1:**
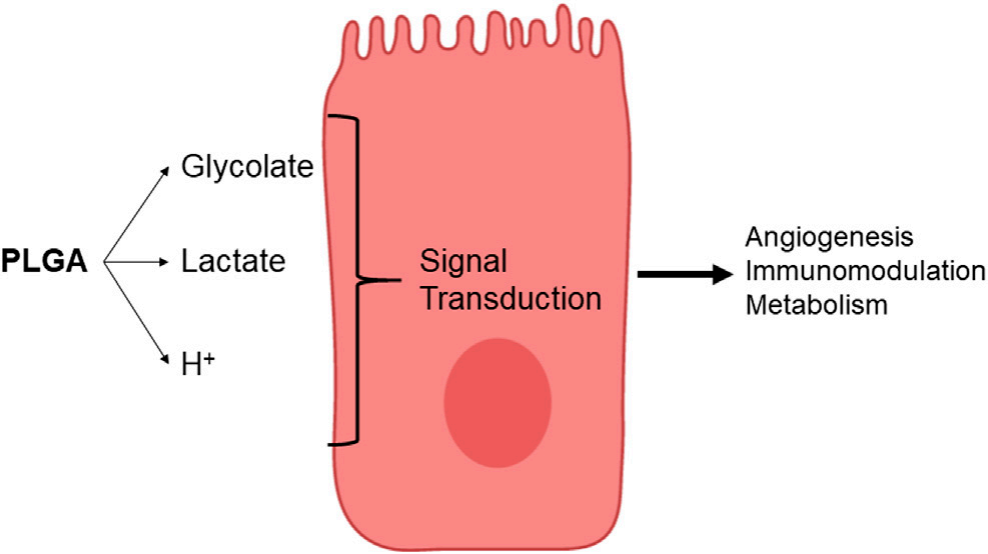
PLGA degradation byproduct. PLGA hydrolysis releases glycolate, lactate and H^+^. Image adapted from Chereddy et al. (2018).

Poly(lactic-co-glycolic acid) (PLGA) has long been known for its high biocompatibility and exceptionally low cytotoxic effects. It is one of the most published on biodegradable polymeric materials used in drug delivery systems and has been able to get endorsements from regulatory bodies, like the US FDA and European Medicine Agency (EMA). PLGA, an aliphatic polyester, has dominated the medical field since its inception in the 1970s, primarily due to its exceptional physicochemical properties and diverse range of biomedical applications (Danhier et al., 2012; Kapoor et al., 2015; Martins et al., 2018). High biocompatibility and low cytotoxicity are attributed to the degradation byproducts—lactate and glycolate—which can easily be incorporated into cellular metabolic pathways (Figure 1) (Chereddy et al., 2018). Due to these highly desirable properties, PLGA has been widely studied as both a therapeutic and diagnostic agent in the cardiac field as well (Oduk et al., 2018; Zhang et al., 2019; Fan et al., 2020a; Fan et al., 2020b). With improvements in processing and production techniques, PLGA has also enjoyed a lot of attention due to the relative ease with which comparatively large batches of nano-medicines can be produced via emulsion polymerization. Using this specific approach, a wide variety of water-soluble and–insoluble loads have been incorporated into PLGA delivery systems (see *Types of Loads Delivered* section for more details).

In recent years, great interest has been shown in polycaprolactone- or PCL-based biomaterials for applications in the biomedical, pharmaceutical, controlled drug delivery, and tissue engineering fields (Kweon et al., 2003; Erndt-Marino et al., 2015; Uto et al., 2016). Even though certain properties, including biocompatibility, biodegradability, mechanical, and structural stability are well-characterized and show great potential, PCL’s lack of bioactivity, due to high hydrophobicity, have resulted in reduced cellular affinity and minimal tissue regeneration rates (Patrício et al., 2013). Approaches to overcome these limitations include the use of nanocomposites, like carbon nanotubes [CNTs (Ho et al., 2017), see *Inorganic Scaffolds* section] as well as co-polymerization with protection-groups like polyethylene glycol (PEG) (Zhou et al., 2016). Viability studies on 3D printed PCL-CNT structures showed that H9C2 myoblasts were able to successfully attach and were healthy for up to 4 days (Ho et al., 2017). Unfortunately, PEGylated PCL nanoparticles did not yield such promising results, as an *in vivo* zebrafish study showed that these particles had a dose-dependent inhibitory effect on angiogenesis, while also upregulating the p53 pro-apoptotic pathway and inducing cellular apoptosis (Zhou et al., 2016).

Among many of the natural polymers that have been investigated as nano-medicines and drug delivery systems (Pereira de Sousa et al., 2015; Mandracchia et al., 2016; Mandracchia et al., 2017), silk is of particular interest, due to its mechanical, physicochemical and biological features (Fu et al., 2009; Crivelli et al., 2018). Silk fibroin (SF), the major constituent of silkworm (*Bombycidae* family) silk, has been an FDA-approved biomaterial since 1993 (Melke et al., 2016). Chen and coworkers found that layer-by-layer (LbL) deposition of chitosan/SF onto nanofibrous patches fabricated via electrospun cellulose nanofibers yielded a 3D micro-environment, leading to enhanced adipose tissue-derived mesenchymal stem cell (AD-MSCs) adherence and engraftment to the epicardium of the infarct-damaged region in rat hearts (Chen et al., 2018). The addition of the SF complemented the impressive mechanical properties demonstrated by the cellulose scaffolds, by making the structures more biocompatible. All *in vivo* assessment and post-operative histology showed that the CS/SF-modified nanofibrous patches promoted the functional survival of the engrafted AD-MSCs and reduced ventricular remodeling post-MI via attenuation of myocardial fibrosis.

Due to their versatile chemistry, interest in polyurethanes (PU) as nano-structured delivery devices and/or targeting agents has been increasing as of late (Mattu et al., 2012; Mattu et al., 2013; Mattu et al., 2015). Unfortunately, very little has been reported with regard to these types of systems with respect to the cardiac field. During the past 5 years, a mere handful of studies have come out. Atorvastatin-loaded PU NPs have been investigated as a potential intravenous route of administration (Eftekhari et al., 2017). Here, fabrication via emulsion-diffusion resulted in NPs of diameter ranging from 174.04 to 277.24 nm, with entrapment efficiencies as high as ∼85% reported. *In vitro* release kinetics showed an 8 day release curve, with both diffusion and polymer-relaxation contributing to the release of the atorvastatin from the PU NPs. Borcan et al. demonstrated that ginger extract could successfully be loaded into PU NPs via spontaneous emulsification (Borcan et al., 2019). The resulting NPs were had a very low water-solubility, and an almost neutral pH, while also being heat resistant up to 280°C. Encapsulation efficiencies as high as 83% were reported, with 60% of the encapsulated ginger extract being released after 5 days.

### Inorganic Scaffolds

Inorganic-based nano-medicines have been of especial interest as diagnostic agents. Magnetic systems, which include superparamagnetic iron oxide nanoparticles (IONs) have shown great promise as an alternative to traditional imaging agents and have gained substantial attention in the past decades (Mahmoudi et al., 2011). These magnetic particles can be utilized as theranostic agents in multimodal imaging facilities including, but not limited to simultaneous magnetic resonance/optical/positron-emission tomography (PET)/single-photon-emission computed tomography (SPECT)/fluorescence imaging (Kim and Judd, 2003; Yen et al., 2013; Bietenbeck et al., 2016; Andrews et al., 2017). Clinical ION-based contrast studies have shown that these particles are not only safe to use, but also demonstrate superior characterization capabilities of myocardial infarct pathology (Alam et al., 2012). It was hypothesized that a major advantage of these particles was their high rate of envelopment by macrophages without envelopment by the peripheral blood monocytes of the study patients (Sosnovik et al., 2007; Yilmaz et al., 2013). In a study with IONs and mesenchymal progenitor cells (MSCs), Han and coworkers found that IONs have the ability to develop the active gap junctional crosstalk of cells like cardiomyoblasts (H9C2) with MSCs for future therapeutic applications (Han et al., 2015). It was found that IONs significantly augmented the expression of the gap junction protein, connexin 43 (Cx43), in the H9C2 cells, which is vital for proper cell-cell communication with MSCs in co-culture. MSCs co-cultured with ION-treated H9C2 showed active cellular crosstalk with the H9C2 cells while also displaying significant increases in electrophysiological cardiac biomarkers along with a paracrine expression profile that was decidedly favorable for cardiac repair, all indicators of this system’s potential for MI repair. Unfortunately, drug delivery via IONs suffers from a number of shortcomings. When conjugating drug molecules to the surfaces or superparamagnetic IONs, systems tend to exhibit reduced drug entrapment efficiencies along with increased failure of drug elution at the target site due to covalent binding. Furthermore, instances of cytotoxicity due to residual concentration of catalysts, like copper, used during the covalent linking of drugs to IONs have been reported, with ION cytotoxicity being reported anywhere between the ranges of 0.1–10 and 100 μg/ml (Ankamwar et al., 2010). These wide ranges in the reported literature strongly suggest that ION cytotoxicity greatly depends on the varying physicochemical characteristics of the particles. Other authors have greatly reduced and even avoided the cytotoxic effects of IONs by coating them with various polymers, including, but not limited to polyvinyl alcohol (PVA) (Mahmoudi et al., 2009a), poly (ethylene glycol)-co-fumarate (PEGF) (Mahmoudi et al., 2009b) and dextrans (Wang et al., 2001).

Nanoscale gold particles (AuNPs) have a wide scope in terms of potential applications in the biomedical world due to their unique biological properties, as anti-oxidative activity and potential to be functionalized as drug delivery systems (Grzelczak et al., 2008; Lewinski et al., 2008; Chandirasekar et al., 2011). PEG coated AuNPs have been shown to be effective mediators of cardiac hypertrophy by attenuating the expression of β-adrenergic receptor levels in mouse models (Qiao et al., 2017). A previous study performed by the same group also showed that the cardiac AuNP content was 6-fold higher in mice undergoing cardiac remodeling than in normal mice. The increased accumulation of AuNPs in the cardiac tissue did not, however, exacerbate isoproterenol-induced cardiac hypertrophy, cardiac fibrosis or cardiac inflammation (Yang et al., 2013). Taken together, these results suggest that AuNPs, especially when modified with a surface coating like PEG, possess exceptional biocompatibility under not only physiological, but also pathological conditions, would likely be safe for cardiac patients and have great translational potential.

Numerous other metal- and metal oxide scaffolds have been investigated as options for cardiac nano-medicines, including but not limited to copper (CuNPs) (Sharma et al., 2018a; Sharma et al., 2018b), cerium oxide (CeO_2_) (Niu et al., 2007), aluminum oxide (Al_2_O_3_) (El-Hussainy et al., 2016), manganese oxide MnO (Zheng et al., 2018) and zinc oxide (ZnO) (Li et al., 2020). Sharma et al. reported that CuNPs showed cardio-protective abilities against ischemia/reperfusion-induced MI (Sharma et al., 2018a; Sharma et al., 2018b). The cardio-protective mechanism was associated with the inhibition of GSK-3 β, with additional improvement noted when the CuNP treatment was combined with exercise preconditioning and training. CeO_2_ NPs have been shown to protect cells in culture from lethal stress, ranging from oxidative stress to radiation-induced stress (Tarnuzzer et al., 2005; Schubert et al., 2006). Niu and colleagues found that intravenous administration of CeO_2_ NP doses as low as 15 nmol twice a week, over a 2 week period, markedly reduced progressive left ventricular (LV) dysfunction and dilatation in their monocyte chemoattractant protein (MCP)-1 mice (Niu et al., 2007). The expression of certain significant endoplasmic reticulum (ER) stress-associated genes, including glucose-regulated protein 78 (Grp78), protein disulfide isomerase (PDI), and heat shock proteins (HSP25, HSP40, HSP70), were suppressed by treatment with CeO_2_ NPs. Although Al_2_O_3_ has been shown to have great antifouling properties, these same characteristics make Al_2_O_3_ a potentially highly cytotoxic material. Studies with Al_2_O_3_ NPs have found that it can lead to myocardial dysfunctions, with variability in myocardial concentrations of nitric oxide (NO), significant decreases in connexin 43 (Cx43) (El-Hussainy et al., 2016). It should be noted that Al_2_O_3_ NP cytotoxicity is highly dependent on concentration as well as crystalline structure (Hashimoto et al., 2016; Nogueira et al., 2019). ZnO NPs, which are widely utilized in the pharmaceutical industry, have been shown to have potential adverse health effects (Jacobsen et al., 2015). A recent study by Li et al. demonstrated that ZnO NPs had both a concentration- and time-dependent cytotoxic effect on in hiPSC-CM (Li et al., 2020). At concentrations, ZnO NOs significantly promoted the generation of reactive oxygen species (ROS) and induced mitochondrial dysfunction. These particles were also noted to impair mitochondrial biogenesis and inhibit the peroxisome proliferator-activated receptor gamma coactivator 1-alpha (PGC-1α) pathway. Additionally, when ZnO NP concentrations were increased they were found to trigger cardiac electrophysiological alterations as evidenced by decreased beating rates and spikes in amplitudes.

**FIGURE 2:**
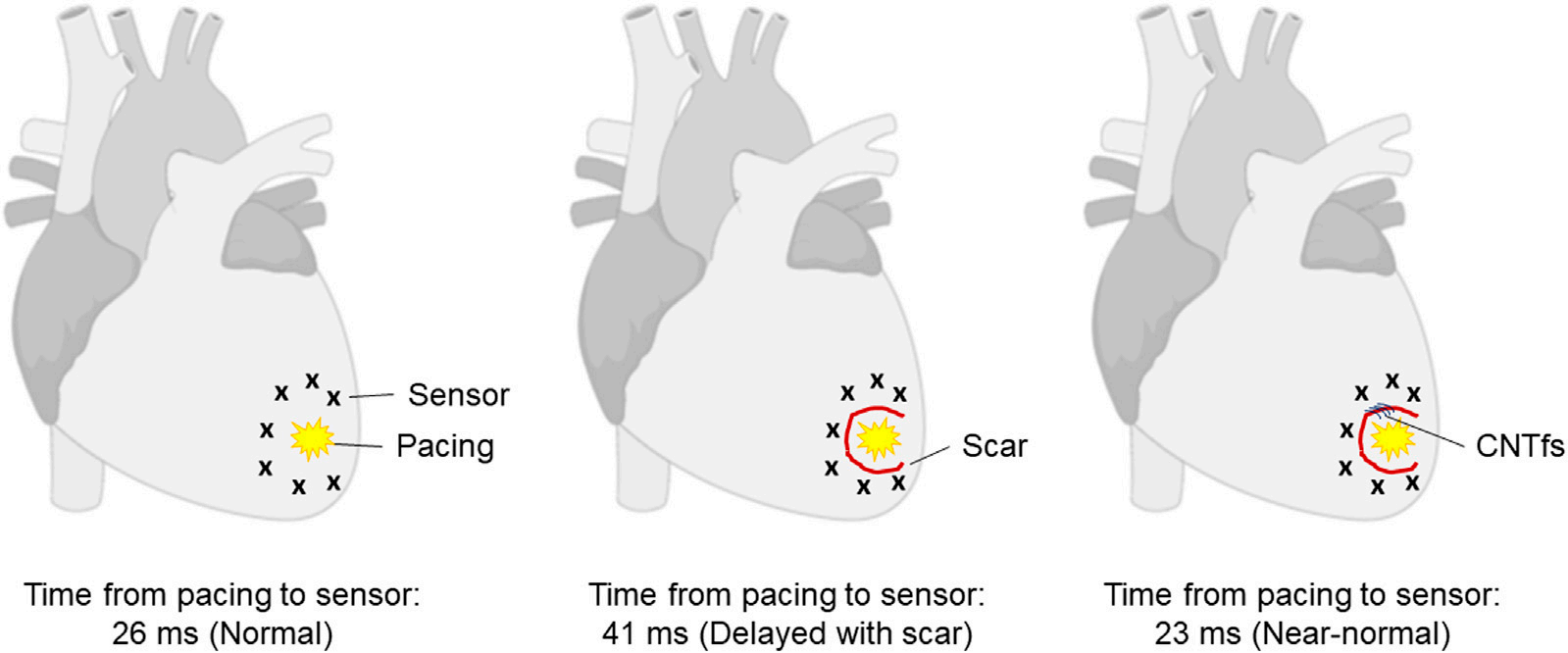
*In vivo* restoration of myocardial conduction with CNTfs. Conductive CNTfs sutured across a blocked area can significantly decrease conduction time to near-normal values. Image adapted from McCauley et al. (2019).

Graphene-based systems, which include carbon nanotubes (CNT), carbon nanotube fibers (CNTf) and graphene oxide (GO) products are of great interest to the biomedical community due to their exceptionally diverse range of chemical and physical properties, which allow for numerous versatile applications. Graphene has both extraordinarily interesting electrical and mechanical properties, combining the conduction properties of a metal with the mechanical strength and stiffness of a polymer fiber with the added benefit of high biocompatibility (Behabtu et al., 2013; Liu et al., 2013; Xu et al., 2016). In the field of cardiac regeneration, re-establishing and facilitating the necessary electrical signaling throughout the damaged tissue are major hurdles still currently faced. When myocytes from neonatal rat ventricles were cultured on substrates with multiwall carbon nanotubes (MWCNTs), they acquired a physiologically more mature phenotype compared to control samples that were cultured on gelatin substrates (Martinelli et al., 2013). It was demonstrated that MWCNT substrates induced the expression of genes associated with terminal differentiation and physiological growth, with a 2-fold increase in α-myosin heavy chain expression (*p* < 0.001) as well as the upregulation of sarcoplasmic reticulum Ca^2+^ ATPase 2a. Single walled carbon nanotubes (SWCNTs) have been employed in the production of a conductive bacterial nanocellulose-based 3D printable biopatch for use in normalization of disrupted cardiac conduction patterns (Pedrotty et al., 2019). These 3D printed patches were shown to not only improve conduction velocities of damaged canine ventricular tissue post-implantation, but to restore them to baseline (∼24–25 cm/s) as noted prior to surgical disruption. CNTfs, with their superior physical properties, have been proposed as an alternative to non-conducting fatigue-resistant fibers used as surgical sutures (McCauley et al., 2019). In this approach, the combined electrical conduction capabilities of the CNTfs, along with their low impedance to ionic charge transfer, biocompatibility, and physiological stability make them ideal candidates that could potentially offer a restorative option while repairing myocardial lesions (Figure 2). It was found that when sewn across the epicardial scar in a sheep model, CNTfs acutely improve conduction. Furthermore, the CNTf/myocardial interface has such low impedance that the CNTfs are able to facilitate the local, downstream myocardial activation.

Research has also shown that these materials are easily functionalized, making them highly useful as theranostic tools (Cai et al., 2003). GO has furthermore been demonstrated to not only be biocompatible, but that it can act as a natural antioxidant to reduce inflammatory polarization of macrophages (M1) via ROS reduction within macrophages (Han et al., 2018). The anti-inflammatory effect of the GO NPs was further enhanced with loading of IL-4 plasmid DNA (IL-4 pDNA) which further polarized M1 macrophages to M2 macrophages leading to significant increases the expression of reparative biomarkers associated with cardiac repair.

## Types of Loads Delivered

Nano-inspired delivery vehicles have been used to encapsulate a plethora of loads in the hope of alleviating the growing burden that various cardiovascular diseases, like ischemic heart injury, place on the medical industry and research fields (Johnson et al., 2014; Jackson et al., 2018; Arnett et al., 2019). Many strategies depend on encouraging remuscularization and/or revascularization of the damaged region, reduction of inflammatory signals or the recruitment of specialized cells via delivery of growth factors, small molecules (chemicals) or exosomes. This section will consider each of these strategies, illuminating the strengths and potential weaknesses of each (see Table 2 for a summary of these systems).

**TABLE 2:** Short summary showing the various nanoparticle systems and their loads, as discussed in this review.

Materials/NP system	Load/Therapeutic	*In vitro/In vivo*	Reference
PLGA NPs	VEGF	*In vitro*: aortic ring bioassay *In vivo*: mouse femoral artery ischemia model	Golub et al. (2010)
PLGA NPs	VEGF	*In vitro*: HUVEC proliferation, tube formation, NP uptake in HUVECs *In vivo*: murine myocardial infarction model	Oduk et al. (2018)
PLGA/PEI NP complexes	IGF-1	*In vitro*: assessment of apoptosis inhibition in freshly isolated CMs *In vivo*: murine myocardial infarction model	Chang et al. (2013)
PLGA NPs	CHIR99021 + FGF1	*In vitro*: assessment of cell cycle progression in vascular cells (ECs and SMCs) *In vivo*: murine myocardial infarction model as well as pig model of IR injury	Fan et al. (2020a)
PLGA NPs	CHIR99021 + FGF1	*In vitro*: assessment of apoptosis inhibition, proliferation and cell cycle activity in hiPSC-CMs *In vivo*: murine myocardial infarction model	Fan et al. (2020b)
PLGA NPs	Pioglitazone	*In vivo*: Mouse and porcine myocardial IR injury model and MI model	Tokutome et al. (2018)
PLGA NPs	TAK-242	*In vivo*: Mouse and myocardial IR injury model	Fujiwara et al. (2019)
PLGA NPs	FK506	*In vivo*: Rat heterotopic heart transplantation model	Deng et al. (2020)
mPEG–PLGA NPs	NO-releasing	*In vitro*: Cytotoxicity assessed on HUVECs, human EP cells, mouse fibroblasts, MCF-7, A549 and C6 cells. Tube formation assay, aortic ring assay	Yang et al. (2018)
Hyaluronan-sulfate NPs	miRNA-21 mimic	*In vivo*: Intravenous administration in a mouse MI model	Bejerano et al. (2018)
RGD-PEG-PLA NPs	microRNA-133	*In vivo*: Rat MI model	Sun et al. (2020)

### Growth Factor Delivery

One of the most widely researched growth factors for cardiac regeneration, specifically due to its vasculogenic properties, is vascular endothelial growth factor (VEGF) (Henry et al., 2001; Sato et al., 2001; Henry et al., 2003; Carmeliet and Jain, 2011; Riley and Smart, 2011). Unfortunately, direct intravenous delivery of VEGF has not yielded any remarkable effects or improvements in preclinical studies (Sato et al., 2001), most likely due to short-lived efficacy and high instability of the protein when injected directly. Intravenous administration of VEGF is further limited by its short *in vivo* half-life (∼30 min) with overall dosages being hampered by off-target site toxicity (Eppler et al., 2002). These hurdles have made encapsulation and entrapment one of the go-to approaches for sustained VEGF delivery. Great interest was shown in producing VEGF PLGA NPs since the early 2000s (Davda and Labhasetwar, 2002; Golub et al., 2010). Golub et al. were able to achieve sustained VEGF release from their ∼400 nm diameter NPs over a 2 week period (Golub et al., 2010). Around 70% of their entrapped VEGF eluted during the first 2 days. Murine aortic ring angiogenesis assays showed significant increases in sprout number with the administration of VEGF-NPs compared to both saline (*p* = 0.001) and empty NPs (*p* < 0.05). Oduk et al. developed VEGF-loaded PLGA NPs with a mean diameter of ∼113 nm using a double emulsion process (Oduk et al., 2018). These NPs showed an encapsulation efficiency of 53.5 ± 1.7% (107.1 ± 3.3 ng VEGF/mg NP), with continuous VEGF release over a 31 day period. *In vivo* studies in murine MI models yielded significant increases in vascular density in the peri-infarct region, reduced infarct sizes, and improvements in LV contractile function 4 weeks post-treatment.

Insulin-like growth factor-(IGF)-1-dependent signaling pathway has been suggested to be involved in cardiac development, acting through the IGF-1 receptor (Wang et al., 1998). IGF-1 is also vital in myocardial function regulation and has been demonstrated to not only promote cardiomyocyte growth but also ensure cardiomyocyte survival. Clinical studies using IGF-1 treatment, have shown that it improves myocardial function post-MI (Duerr et al., 1995; Donath et al., 1998). Unfortunately, extended IGF-1 overexpression has been shown to reduce cardiac functional recovery post-MI, making controlled delivery vital for its future use as a cardiac medicine (Prêle et al., 2012). Chang and coworkers developed PLGA-IGF-1 complexed NPs of different sizes—60 nm, 200 nm, and 1 μm specifically (Chang et al., 2013). Following MI in murine models, the NPs were administered via intra-myocardial injection. It was found that PLGA-IGF-1 NP treatment allowed for retention of significantly more IGF-1 in the myocardium than IGF-1 free-drug treatment at 2, 6, 8, and 24 h respectively. Most importantly, it was noted that a single intra-myocardial injection of the PLGA-IGF-1 NPs was sufficient to prevent cardiomyocyte apoptosis (*p* < 0.001), reduce infarct sizes (*p* < 0.05), and improve LV ejection fraction (*p* < 0.01) 21 days post-MI.

Recent studies showing the synergistic effects of using a combination of extended delivery of CHIR99021 (a Wnt1 agonist/GSK-3β antagonist) and fibroblast growth factor 1 (FGF1) to protect ischemia-threatened cardiomyocytes from apoptosis, while accelerating angiogenesis through the promotion of endothelial and vascular SMC proliferation, and consequently enhance myocardial protection have been published (Fan et al., 2020a; Fan et al., 2020b). These studies found that PLGA NPs loaded with CHIR99021 or FGF1 allowed for effective delayed release for up to 4 weeks. Intra-myocardial injection of these NPs enabled myocardial protection and reduced infarct sizes by 20–30% in murine or porcine models of post-MI LV remodeling. The combination of CHIR and FGF1 was also found to promote cell cycle progression.

### Small Molecule Delivery

A variety of small molecules have been loaded into nano-delivery systems for numerous purposes, ranging from enticing angiogenic responses, to preventing cardiac allograft rejection via altering inflammatory responses (Giannouli et al., 2018; Tokutome et al., 2018; Yang et al., 2018; Fujiwara et al., 2019; Deng et al., 2020). Monocyte-mediated inflammation is one of the major issues faced during myocardial ischemia–reperfusion (IR) injury as well as the healing process following acute myocardial infarction (AMI). Tokutome et al. found that pioglitazone, a peroxisome proliferator-activated receptor-gamma (PPARγ) agonist, had unique anti-inflammatory effects on monocytes/macrophages and when administered via a targeted NP approach, it had the potential to ameliorate IR injury and cardiac remodeling in preclinical animal models (Tokutome et al., 2018). Fujiwara and coworkers developed PLGA NPs containing TAK-242 (TAK-242-NP), a chemical inhibitor of Toll-like receptor 4 (TLR4) intracellular domain. Intravenous administration of TAK-242-NP (1.0 or 3.0 mg/kg TAK-242-NP) at the time-of-reperfusion reduced infarct sizes in wild type murine models (Fujiwara et al., 2019). Additional studies were performed with TLR4-deficient mice to eliminate the possibility that TAK-242-NP reduced infarct sized via TLR4-independent mechanisms. Immunosuppressive agents, such as FK506, greatly reduce chances of allograft rejection. Although FK506 has shown a high efficiency, its long-term systemic administration inevitably induces side-effects, including but not limited to nephrotoxicity, neurotoxicity, hypertension and diabetogenic effects. Recently, a rat heterotopic heart transplantation model was established to determine the therapeutic efficacy and potential effects of PLGA NPs loaded with FK506 (FK506-NPs), which were prepared via an emulsion solvent evaporation method (Deng et al., 2020). FK506-NPs not only successfully alleviated acute allograft rejection, but also prolonged graft survival compared with free FK506 treatment (mean survival time, 17.1 ± 2.0 vs. 13.3 ± 1.7 days).

Nitric oxide (NO) is known to induce multiple biological functions by stimulating cellular signaling pathways. Some NO-driven functions include various human physiological processes, such as immune responses, inhibition of platelet aggregation, angiogenesis and apoptosis (Seabra et al., 2015). Of special interest in the clinical milieu is the angiogenic activity of NO and its potential for repairing or regenerating damaged tissue specifically caused by the degradation of the extracellular matrix (Clapp et al., 2009). Issues with NO’s short half-life have been skirted by using NO donor molecules, including but not limited N-diazeniumdiolate (NONOate) and S-nitrosothiol (RSNO) for example. Yang et al. developed methoxy PEG-PLGA (mPEG-PLGA) NO-releasing NPs, via diethylenetriamine NONOate entrapment (Yang et al., 2018). *In vitro* tube formation assays confirmed the angiogenic potential of the NO-releasing NPs, while aorta ring assays were used *ex vivo*. Tubular formation increased 189.8% in NO-NP–treated groups compared with that in the control group, while rat aorta demonstrated vigorous sprouting angiogenesis in response to NO-NPs.

### Exosome Delivery and “Synthetic” Exosome Design

In the field of cardiovascular medicine, specifically the area related to treatment via implanted cells and structures, a lot of speculation remains regarding the extent of the effects that paracrine signaling has on the repair process (Gnecchi et al., 2005). Recent studies have shown that many of the therapeutic potential of MSCs and human induced pluripotent stem cells (hiPSCs) for example can be largely attributed to exosomes, a specific type of extracellular vesicle (EVs). These EVs have diameters ranging between 50 and 200 nm, contain proteins and RNAs, and are involved in intercellular communications by acting as carriers of bioactive molecules (Lai et al., 2010; Beltrami et al., 2017; Gao et al., 2020). The therapeutic potential of exosomal delivery and treatment derives from the fact that RNAs and proteins in the exosomes transferred from the parental cells to the recipient cells are functional in the recipient cells and participate in the regulation of intracellular signaling cascades (Valadi et al., 2007; Bang and Thum, 2012). Unfortunately, the clinical therapeutic potential of exosomes are greatly hampered by production capability limitations relative to the amount required for patient treatment (Dai et al., 2008; Katsuda et al., 2013). The idea of creating an exosome-mimetic nano-vesicle or a “synthetic” exosome is thus a very attractive prospect from a clinical point of view.

Bejerano et al. showed that the delivery of a miRNA-21 mimic with hyaluronan-sulfate NPs in macrophage-enriched areas of the infarcted heart could induce a phenotypic switch, from pro-inflammatory to reparative (Bejerano et al., 2018). Following intravenous administration in a mouse MI model, the miRNA-21 NPs targeted cardiac macrophages at the infarct zone, induced their phenotype switch from pro-inflammatory to reparative, promoted angiogenesis, while also reducing hypertrophy, fibrosis and cell apoptosis in the remote region of the myocardium.

Following left anterior descending (LAD) coronary artery ligation in rats, Sun et al. administered PEG-PLGA NPs modified with arginine-glycine-aspartic acid tripeptide (RGD), loaded with microRNA-133 via tail vein injections (Sun et al., 2020). The RGD-PEG-PLA NPs were able to target the infarcted hearts, while improving cardiac histopathological changes, reducing the apoptotic effects on cardiomyocytes, and decreasing the expression levels of factors associated with myocardial injury. It was postulated that the regulation of the SIRT3/AMPK pathway was involved in the protective role that the NPs played.

## Conclusion

Nano-medicines have shown great promise, not only as therapeutic agents, but as diagnostic agents as well. Recently great interest has been shown in combining these aspects into theranostic applications, which would allow for less invasive and more effective treatment of patients in the future. Even though many *in vivo* studies have shown great promise with their optimized nano-medicine systems, wash-out of the particles still remains an ever-present limitation, which may be overcome by combining NP delivery with tissue engineering approaches, including but not limited to polymer scaffolds or cardiac muscle patches which can be implanted in the damaged region for example. Further advances have been made with new delivery methods, including inhalation, surpassing previous needs for painful implantation procedures or injections (Miragoli et al., 2018). With advancements made in fields including chemistry, physics and material engineering, nano-medicines are expected to become more commonplace in the cardiac field in the future.
